# Supplementary material for: The effects of genital myiasis on the diversity of the vaginal microbiota in female Bactrian camels
Source: BMC Vet Res. 2022 Mar 5;18:87. doi: 10.1186/s12917-022-03189-5 (PMC8897907; doi:10.1186/s12917-022-03189-5)
Supplement: Supplementary file 5 — Additional file 5. [file 12917_2022_3189_MOESM5_ESM.zip › MPL201709200_16s_yy/Treat1/css/js_css/jsPDF-master.zip!/jsPDF-master/docs/examples_js_test_harness.js.html]

examples/js/test\_harness.js - Documentation


## Home

### Classes

- FontObject
- jsPDF
- PubSub

### Global

- addFont
- addMetadata
- addPage
- CapJoinStyles
- circle
- CssColors
- ellipse
- getFontList
- html2pdf
- init
- jsPDFEditor
- lines
- output
- pdf\_test\_harness\_init
- rect
- requirejs
- reset
- roundedRect
- save
- setDrawColor
- setFillColor
- setFont
- setFontSize
- setFontStyle
- setLineCap
- setLineJoin
- setLineWidth
- setProperties
- setTextColor
- text
- toUrl
- triangle
- triggerEvent
- update

# examples/js/test\_harness.js

```
/**
 * jsPDF PDF Test Harness
 * Copyright (c) 2014 Steven Spungin (TwelveTone LLC)  steven@twelvetone.tv
 *
 * Licensed under the MIT License.
 * http://opensource.org/licenses/mit-license
 */ 

/**
 * An easy way to view PDF and PDF source code side by side.
 */
pdf_test_harness_init = function(pdf, message) {

	var harness = new pdf_test_harness();

	var body = document.getElementsByTagName('body')[0];
	body.style.display = 'flex';

	var div = document.createElement('div');
	div.setAttribute('style', 'position:fixed;height:20px;left:0;right:0;background:lightblue');
	body.appendChild(div);
	harness.header = div;

	var div2 = document.createElement('div');
	div2.setAttribute('style', 'position:fixed;display:flex;top:20px; bottom:0;left:0;right:0');
	body.appendChild(div2);
	harness.body = div2;

	var btn1 = document.createElement('input');
	btn1.setAttribute('type', 'radio');
	btn1.setAttribute('name', 'view');
	div.appendChild(btn1);
	btn1.checked = true;

	var lbl1 = document.createElement('label');
	lbl1.setAttribute('for', 'btn1');
	lbl1.innerHTML = 'PDF'
	div.appendChild(lbl1);

	var btn2 = document.createElement('input');
	btn2.setAttribute('type', 'radio');
	btn2.setAttribute('name', 'view');
	div.appendChild(btn2);

	var lbl2 = document.createElement('label');
	lbl2.setAttribute('for', 'btn2');
	lbl2.innerHTML = 'Source'
	div.appendChild(lbl2);

	var btn3 = document.createElement('input');
	btn3.setAttribute('type', 'radio');
	btn3.setAttribute('name', 'view');
	div.appendChild(btn3);

	var lbl3 = document.createElement('label');
	lbl3.setAttribute('for', 'btn3');
	lbl3.innerHTML = 'Both'
	div.appendChild(lbl3);

	harness.source = document.createElement('pre');
	harness.source.setAttribute('style', 'margin-top:0;width:100%;height:100%;position:absolute;top:0px;bottom:0px;overflow:auto');
	div2.appendChild(harness.source);

	harness.iframe = document.createElement('iframe');
	harness.iframe.setAttribute('style', 'width:100%;height:100%;position:absolute;overflow:auto;top:0px;bottom:0px');
	div2.appendChild(harness.iframe);

	//if (pdf_test_harness.onload) {
	//harness.pdf = pdf_test_harness.onload(harness);
	if (message) {
		message += "<p style='text-align:center;font-style:italic;font-size:.8em'>click to close</p>";
		var popup = document.createElement('div');
		popup.setAttribute('style', 'z-index:100;margin:100px auto;cursor:pointer;font-size:1.3em;top:50px;background-color:rgb(243, 224, 141);padding:1em;border:1px solid black');
		popup.innerHTML = message;
		body.appendChild(popup);
		popup.onclick = function() {
			popup.parentNode.removeChild(popup);
		}
	}
	//}

	harness.pdf = pdf;
	harness.render('pdf');

	btn1.onclick = function() {
		harness.render('pdf');
	}
	btn2.onclick = function() {
		harness.render('source');
	}
	btn3.onclick = function() {
		harness.render('both');
	}
	
	return harness;
}

pdf_test_harness = function(pdf) {
	this.pdf = pdf;
	this.onload = undefined;
	this.iframe = undefined;

	this.entityMap = {
		"&" : "&amp;",
		"<" : "&lt;",
		">" : "&gt;",
		'"' : '&quot;',
		"'" : '&#39;',
		"/" : '&#x2F;'
	};

	this.escapeHtml = function(string) {
		return String(string).replace(/[&<>"'\/]/g, function(s) {
			return this.entityMap[s];
		}.bind(this));
	};

	this.getParameterByName = function(name) {
		name = name.replace(/[\[]/, "\\[").replace(/[\]]/, "\\]");
		var regex = new RegExp("[\\?&]" + name + "=([^&#]*)"), results = regex.exec(location.search);
		return results === null ? "" : decodeURIComponent(results[1].replace(/\+/g, " "));
	};

	this.setPdf = function(pdf) {
		this.pdf = pdf;
		this.rendered = undefined;
		this.render(this.view);
	};

	// generate the pdf, the source code, or both
	this.render = function(view) {
		this.view = view;
		//Current code only lets us render one time.
		if (!this.rendered) {
			this.rendered = this.pdf.output('datauristring');
			this.iframe.src = this.rendered;
			var raw = this.pdf.output();
			raw = this.escapeHtml(raw);
			this.source.innerHTML = raw;
		}
		if ('pdf' === view) {
			this.source.style.display = 'none';
			this.iframe.style.display = 'block';
			this.iframe.style.width = '100%';
		} else if ('source' === view) {
			this.iframe.style.display = 'none';
			this.source.style.display = 'block';
			this.source.style.width = '100%';
		}

		if ('both' === view) {
			raw = this.escapeHtml(raw);
			this.iframe.style.width = '50%';
			this.iframe.style.position = 'relative';
			this.iframe.style.display = 'inline-block';
			this.source.style.width = '50%';
			this.source.style.position = 'relative';
			this.source.style.display = 'inline-block';
		}
	}
}
```

  

Documentation generated by JSDoc 3.4.1 on Mon Oct 03 2016 12:36:18 GMT+0100 (BST) using the docdash theme.
